# Monthly runoff prediction based on a coupled VMD-SSA-BiLSTM model

**DOI:** 10.1038/s41598-023-39606-4

**Published:** 2023-08-12

**Authors:** Xianqi Zhang, Xin Wang, Haiyang Li, Shifeng Sun, Fang Liu

**Affiliations:** 1 Water Conservancy College, North China University of Water Resources and Electric Power, Zhengzhou, 450046 China; 2 Collaborative Innovation Center of Water Resources Efficient Utilization and Protection Engineering, Zhengzhou, 450046 China; 3 Technology Research Center of Water Conservancy and Marine Traffic Engineering, Henan Province, Zhengzhou, 450046 China

**Keywords:** Environmental sciences, Hydrology, Natural hazards

## Abstract

The accurate prediction of monthly runoff in the lower reaches of the Yellow River is crucial for the rational utilization of regional water resources, optimal allocation, and flood prevention. This study proposes a VMD-SSA-BiLSTM coupled model for monthly runoff volume prediction, which combines the advantages of Variational Modal Decomposition (VMD) for signal decomposition and preprocessing, Sparrow Search Algorithm (SSA) for BiLSTM model parameter optimization, and Bi-directional Long and Short-Term Memory Neural Network (BiLSTM) for exploiting the bi-directional linkage and advanced characteristics of the runoff process. The proposed model was applied to predict monthly runoff at GaoCun hydrological station in the lower Yellow River. The results demonstrate that the VMD-SSA-BiLSTM model outperforms both the BiLSTM model and the VMD-BiLSTM model in terms of prediction accuracy during both the training and validation periods. The Root-mean-square deviation of VMD-SSA-BiLSTM model is 30.6601, which is 242.5124 and 39.9835 lower compared to the BiLSTM model and the VMD-BiLSTM model respectively; the mean absolute percentage error is 5.6832%, which is 35.5937% and 6.3856% lower compared to the other two models, respectively; the mean absolute error was 19.8992, which decreased by 136.7288 and 25.7274 respectively; the square of the correlation coefficient (*R*^*2*^) is 0.93775, which increases by 0.53059 and 0.14739 respectively; the Nash–Sutcliffe efficiency coefficient was 0.9886, which increased by 0.4994 and 0.1122 respectively. In conclusion, the proposed VMD-SSA-BiLSTM model, utilizing the sparrow search algorithm and bidirectional long and short-term memory neural network, enhances the smoothness of the monthly runoff series and improves the accuracy of point predictions. This model holds promise for the effective prediction of monthly runoff in the lower Yellow River.

## Introduction

Runoff simulation and prediction play a vital role in water resource management, regulation, and rational planning. They are crucial for the efficient utilization of water resources, flood control, and disaster reduction. With the rapid advancement of artificial intelligence technology, numerous deep learning algorithms have emerged, and comprehensive forecasting models based on intelligent methods and numerical weather prediction have been proposed. These models involve various optimization algorithms such as Chaos Optimization Algorithm^1^, bald eagle search optimization algorithm^2^, Particle Swarm Optimization (PSO)^3^, and artificial neural network models^4,5^, which have deepened their intersectionality with hydrology.

The pursuit of a runoff prediction model with high accuracy and applicability has been a topic of constant concern in hydrological forecasting. Both domestic and foreign researchers have conducted extensive studies to improve the accuracy of prediction models, resulting in fruitful results. For instance, Feng et al.^6^ combined quantum behavioural particle swarm optimization algorithms with variational modal decomposition and SVM to build a monthly runoff prediction model. Lei et al.^7^ proposed a prediction model that combines empirical mode decomposition and Metropolis Hastings sampling Bayesian model for hydrological prediction. Roy^8^ used the LSTM model to predict ET0 in multiple watersheds with daily and multi-step forward predictions. The LSTM model demonstrated strong adaptability to various prediction indicators. Xu Dongmei et al.^9^ utilized LSSVM and CEEMDAN to predict monthly runoff at Changshui hydrological station. Fan Hongxiang and others developed a meteorological runoff model for the Poyang Lake basin based on the Long short-term memory neural network, effectively simulating the runoff process, capturing extreme runoff values, and reflecting short-term fluctuations. The Long Short Term Memory (LSTM) neural network model has been widely employed in runoff prediction due to its nonlinear prediction capability, faster convergence speed, and long-term memory effect. However, when the LSTM model learns time series, it faces challenges such as a poor early feature memory effect, leading to the loss of features in the initial learned part, and difficulties in fully capturing the entire time series features^10^.

As research progresses, there is an increasing demand for higher accuracy in runoff prediction. Seo^11^ and He^12^ developed a combined runoff prediction model based on the VMD algorithm, demonstrating improved prediction accuracy after data decomposition and reconstruction. Ba et al.^13^ used the sparrow search algorithm (SSA) to optimize artificial neural networks (ANN model), showcasing the advantages of simple implementation, high search accuracy, fast convergence speed, stability, and robustness. Chen and Liang^14^ combined the empirical modal decomposition method (EMD), attention mechanism, and BiLSTM neural network to predict the daily longitudinal flow at the Qingxi River site in Xuanhan County, obtaining accurate results that met prediction requirements. Regarding the study of bidirectional long short-term memory neural networks, Li and Jiang^15^ used STL for sequence decomposition, temporal convolution, a bidirectional long short-term memory network (TCN-BiLSTM) for feature leaning the decomposed series, and interdependent moment feature emphasisation using DMAttention to predict the concentration of air pollutants, achieving accurate prediction accuracy. Sathi et al.^16^ used a CNN-BiLSTM model to predict the attention to the induced electric field of a transcranial magnetic stimulation coil with similarly excellent results.Current research on monthly runoff prediction models, both domestically and internationally, mainly focuses on coupling optimization algorithms with neural networks to improve prediction accuracy by constructing coupled models. Overall, as the complexity of hydrological phenomena deepens, the development of conceptual and physical hydrological models encounters certain bottlenecks^17^, and the accuracy of predictions needs further improvement. Therefore, this article proposes a VMD-SSA-BiLSTM coupled model suitable for predicting monthly runoff in the lower Yellow River. The model combines the variational mode decomposition (VMD) used for signal decomposition and preprocessing, the sparse search algorithm (SSA) used for parameter optimization of the BiLSTM model, and the bidirectional Long short-term memory neural network (BiLSTM) used to utilize the bidirectional links and advanced characteristics of the runoff process. The proposed VMD SSA-BiLSTM model combines the sparrow search algorithm and the bidirectional Long short-term memory neural network, which enhances the smoothness of monthly runoff series and improves the accuracy of point prediction.

## Theory and method

### Sparrow Search Algorithm (SSA)

The Sparrow Search Algorithm (SSA) is an intelligent optimization algorithm that draws inspiration from the foraging and anti-predation behavior of sparrows^18^. The optimization process of SSA can be described as follows:

The formula for updating the discoverer's position $X_{i,\;j}^{t + 1}$ is:

1 $$X_{i,\;j}^{t + 1} = \left\{ {\begin{array}{*{20}l} {X_{i,\;j}^{t + 1} \exp \left[ { - i/\alpha i_{{ter_{max} }} } \right]} \hfill & {R_{2} < S_{T} } \hfill \\ {X_{i ,\;j}^{t} + QL} \hfill & {R_{2} \ge S_{T} } \hfill \\ \end{array} } \right.$$

where $i_{{ter_{max} }}$ is the maximum number of iterations, $x_{i,j}$ represents the position of the *i*-th sparrow in the *j*-th dimension, *α* is a random number in the range (0, 1], *R*_2_ is the early warning value in the range [0, 1], *S*_*T*_ is the safe value in the range [0.5, 1], *Q* is a random number following a normal distribution, and *L* is a 1 × d matrix with all elements set to 1.

Additionally, the joiner location $X_{i,\;j}^{t + 1}$ is updated using a specific process:

2 $$X_{i,\;j}^{t + 1} = \left\{ {\begin{array}{*{20}l} {Q\exp [\left( {X_{worst} - X_{i,\;j}^{t} } \right)/i^{2} } \hfill & {i > n/2} \hfill \\ {X_{p}^{t + 1} + \left| {X_{i,\;j}^{t} - } \right.\left. {X_{p}^{t + 1} } \right|A^{ + } L} \hfill & {Others} \hfill \\ \end{array} } \right.$$

In equation $A^{ + } = A^{T} (AA^{T} )^{ - 1}$, $X_{p}$ is the optimal position of the current discoverer; ${X}_{worst}$ is the current global worst position, and *A* is a 1 × d matrix with randomly assigned elements of either 1 or − 1.

Furthermore, it is assumed that a certain percentage (10–20%) of the sparrow flock are aware of the danger^19^. These vigilant sparrows swiftly move towards the safety zone, and the mathematical expression for the location of the vigilantes $X_{i,j}^{t + 1}$ is:

3 $$X_{i,j}^{t + 1} = \left\{ {\begin{array}{*{20}l} {X_{best}^{t} + \beta \left| {X_{i,\;j}^{t} - X_{best}^{t} } \right|} \hfill & {f_{i} > f_{g} } \hfill \\ {X_{i,\;j}^{t} + K\left( {\frac{{\left| {\left. {X_{i,j}^{t} - X_{worst}^{t} } \right|} \right.}}{{\left( {f_{i} - f_{w} } \right) + \varepsilon }}} \right)} \hfill & {f_{i} = f_{g} } \hfill \\ \end{array} } \right.$$

In the formula, $X_{best}$ is the current global optimal position, *β* is a random number used as a step control parameter following a normal distribution with a mean of 0 and a variance of 1, *K* is a random number within the range [− 1, 1], ${f}_{i}$ is the fitness value of the individual sparrow, ${f}_{g}$ is the current global optimal fitness value, ${f}_{w}$ is the current global worst adaptation value, and *ℇ* is a constant^20^.

The algorithmic steps of the Sparrow Search Algorithm (SSA)^21^ are as follows:

- *Step 1* Initialize the population, specify the proportion of predators and joiners, and set the number of iterations.
- *Step 2* Calculate the fitness values for each sparrow and sort them in descending order.
- *Step 3* Update the positions of the discoverers.
- *Step 4* Update the positions of the joiners.
- *Step 5* Update the positions of the vigilant sparrows (those aware of danger).
- *Step 6* Calculate the fitness values and update the positions of the sparrows.
- *Step 7* If the desired requirements are met, output the result; otherwise, repeat steps 2 to 6.

**Figure 1:**
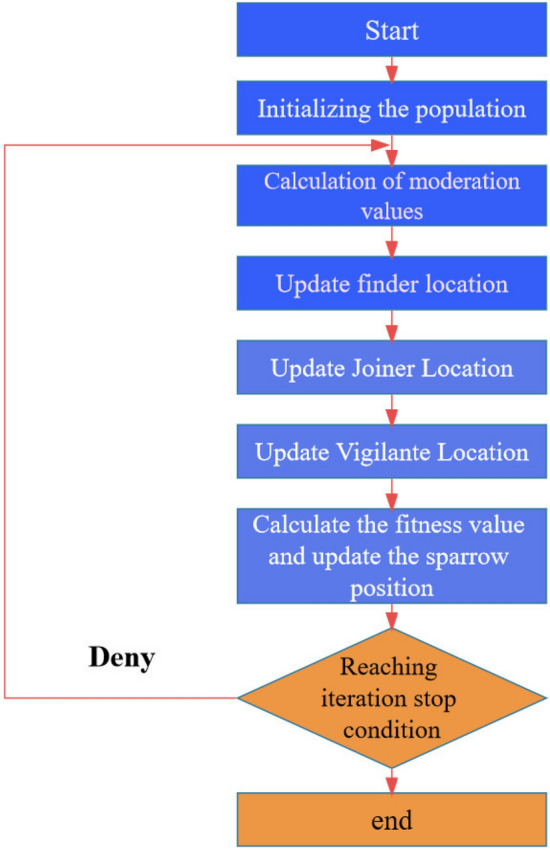
Sparrow search algorithm optimization flowchart.

The flow chart illustrating the Sparrow Search Algorithm is depicted in Fig. 1.

In this study, the Sparrow Search Algorithm (SSA) is employed to optimize three key parameters of the BiLSTM model: the number of hidden units, the maximum training period, and the initial learning rate.

### Variational mode decomposition (VMD)

Variational Modal Decomposition (VMD) is a non-recursive decomposition model that employs an adaptive variational method to determine frequency bands and estimate corresponding modes, effectively balancing the errors between them^22^. VMD aims to decompose a given real input signal *f*(*t*) into discrete sub-signals or modes, denoted as *μk*, where each mode *uk* is primarily concentrated around a center frequency *wk*^23^. The specific steps for VMD to decompose *f*(*t*) into *k* subsequences^24^ are as follows:

- *Step 1* Calculate the analytic signal and construct the spectrum for each mode *μk* using the Hilbert transform.
- *Step 2* Shift the frequency spectrum of the modes to the baseband by utilizing their respective estimated center frequencies.
- *Step 3* Estimate the bandwidth by demodulating the Gaussian smoothness of the signal, which is represented by the *L*_*2*_ norm of the gradient.

This results in a constrained variational problem, which can be expressed as follows:

4 $$\left\{\begin{array}{c}\genfrac{}{}{0pt}{}{min}{\left\{uk\right\},\left\{wk\right\}}\left\{\sum_{k}{\Vert \partial \left(t\right)\left[\left(\delta \left(t\right)+\frac{j}{\pi t}\right)uk\left(t\right)\right]{e}^{-jwkt}\Vert }_{2}^{2}\right\}\\ s.t.\sum_{k}uk=f\end{array}\right.$$

In the formula, ${u}_{k}$ is the k-th modal component, ${w}_{k}$ is the frequency center associated with the *k*-th mode, and ${\delta }_{(t)}$ is the unit pulse function. To handle the constrained variational problem, quadratic penalty terms and Lagrange multiplier $\lambda$ are introduced to transform it into an unconstrained problem. The alternating direction multiplier method (ADMM) is then employed for solving the transformed problem^25^. In this study, the VMD technique is applied to decompose the monthly runoff time series into a set of relatively smooth sub-series data. This decomposition helps enhance the prediction accuracy of the model.

### Bidirectional long short-term memory network (BiLSTM)

**Figure 2:**
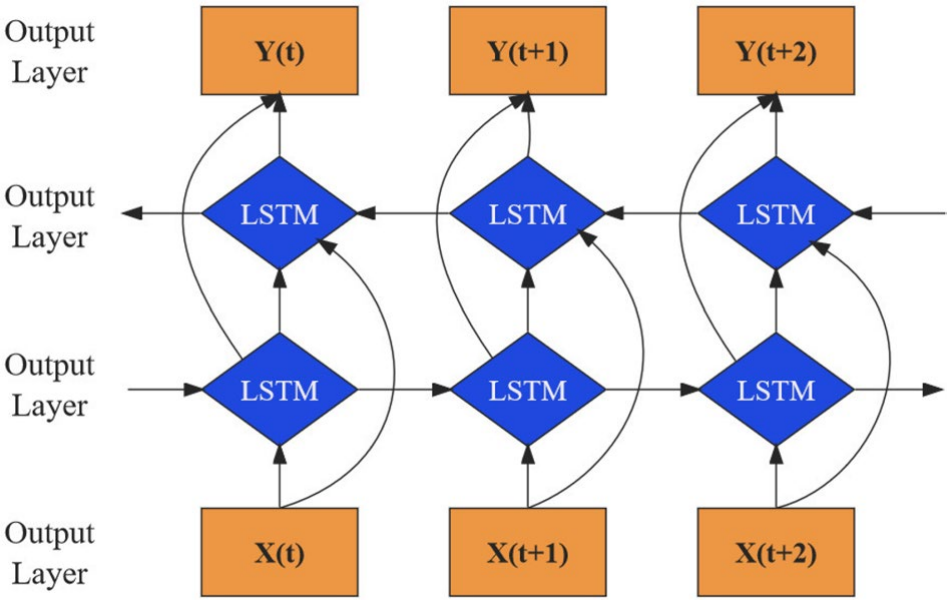
Construction of bidirectional long short-term memory neural network.

Bidirectional Long Short-Term Memory (BiLSTM) is an advanced variation of traditional bidirectional recurrent neural networks that replaces regular RNN units with LSTM units. It consists of both forward and backward LSTM components^26^. By incorporating information from both past and future contexts of a sequence, BiLSTM effectively captures a comprehensive range of features. The hidden layer of BiLSTM consists of two parts: the forward LSTM cell state and the backward LSTM cell state. Historical sequences are fed into the hidden layer through the input layer, enabling forward and backward computations. The model learns from the past and future sequence features to produce the final output result^27^. The network structure of BiLSTM, as illustrated in Fig. 2, where $X(t)$ represents the input of the network and $Y(t)$ represents the output of the network^21^.

### SSA optimization BiLSTM

To obtain optimal parameters for model prediction, the Sparrow Search Algorithm (SSA) was employed to optimize the number of hidden units, the maximum training period, and the initial learning rate of the Bidirectional Long Short-Term Memory (BiLSTM) network. The construction of the SSA-BiLSTM model involved the following steps:

- *Step 1* The dataset was divided into training and testing sets, which were then normalized.
- *Step 2* The training set was used as the input vector for training the BiLSTM network.
- *Step 3* The Sparrow Search Algorithm was applied to optimize the number of hidden units, the maximum training cycle, and the initial learning rate of the BiLSTM model, resulting in the identification of optimal parameters. These parameters were selected to establish the model.
- *Step 4* The optimized BiLSTM model was trained again, and the results were compared.
- *Step 5* Termination conditions were evaluated. If met, the loop was exited and the prediction results were outputted. Otherwise, the process was repeated.
- *Step 6* The test set was fed into the optimized BiLSTM model, and the prediction results were obtained.

**Figure 3:**
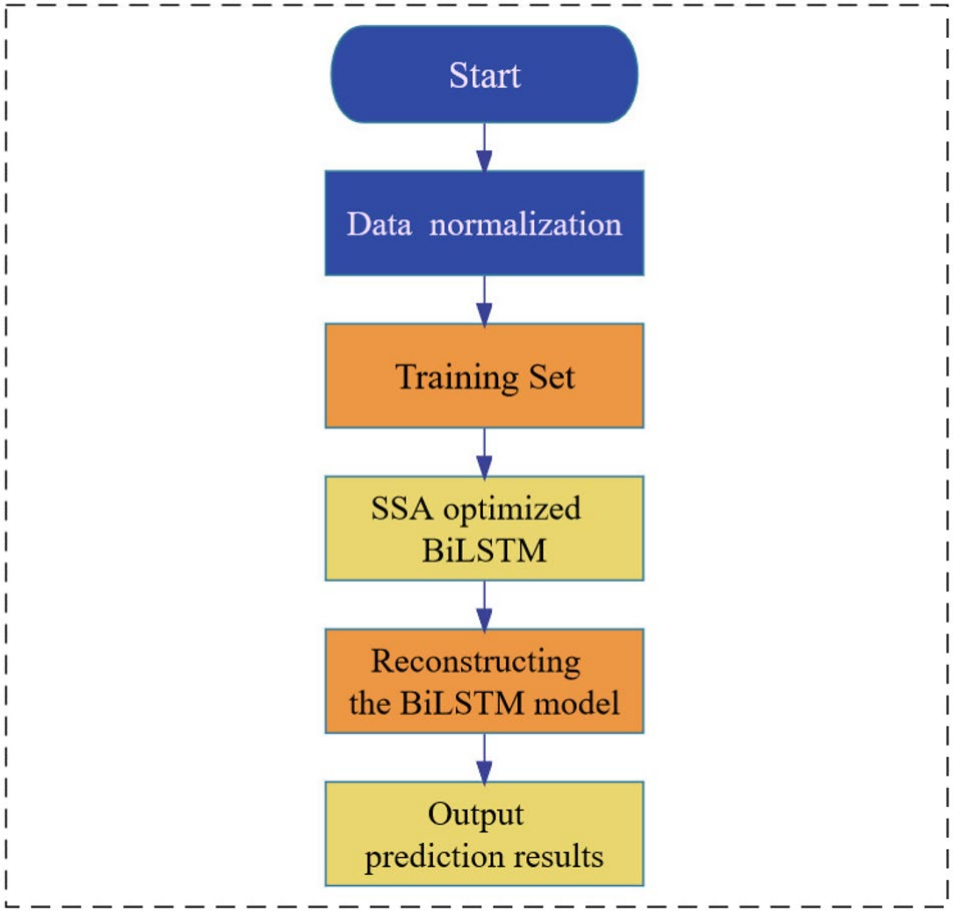
Flow chart of SSA optimized BiLSTM model.

The corresponding flow chart depicting these steps is illustrated in Fig. 3.

### VMD-SSA-BiLSTM model prediction process

The VMD-SSA-BiLSTM model is a combination of Variational Modal Decomposition (VMD), Sparrow Search Algorithm (SSA), and Bidirectional Long Short-Term Memory (BiLSTM) networks. The prediction steps for monthly runoff using this model are as follows:

- *Step 1* Select the monthly runoff data from the previous n months as input to the model.
- *Step 2* Decompose the original runoff sequence using VMD to obtain k components.
- *Step 3* Define the search range for the sparrow population size *N*, maximum iteration number *M*, and parameter range. Then, establish the coupled model of SSA and BiLSTM by selecting Mean Squared Error (*MSE*) as the objective function in the optimization algorithm.
- *Step 4* Input each component separately into the SSA-BiLSTM prediction models to obtain *k* individual prediction models.
- *Step 5* Sum up the predicted values from the k prediction models to obtain the final predicted values for monthly runoff.

**Figure 4:**
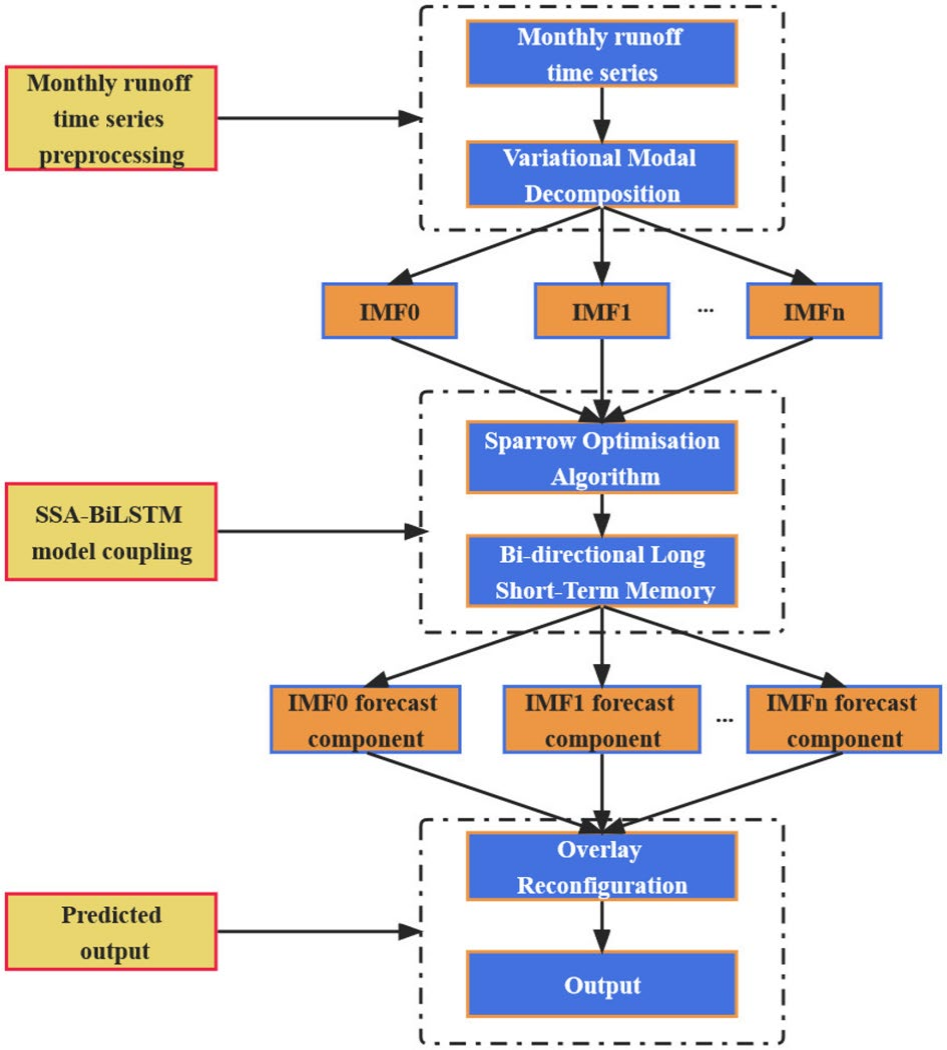
VMD-SSA-BiLSTM model flowchart.

The flow chart depicting the construction of the VMD-SSA-BiLSTM model is presented in Fig. 4.

## Example applications

### Data sources

**Figure 5:**
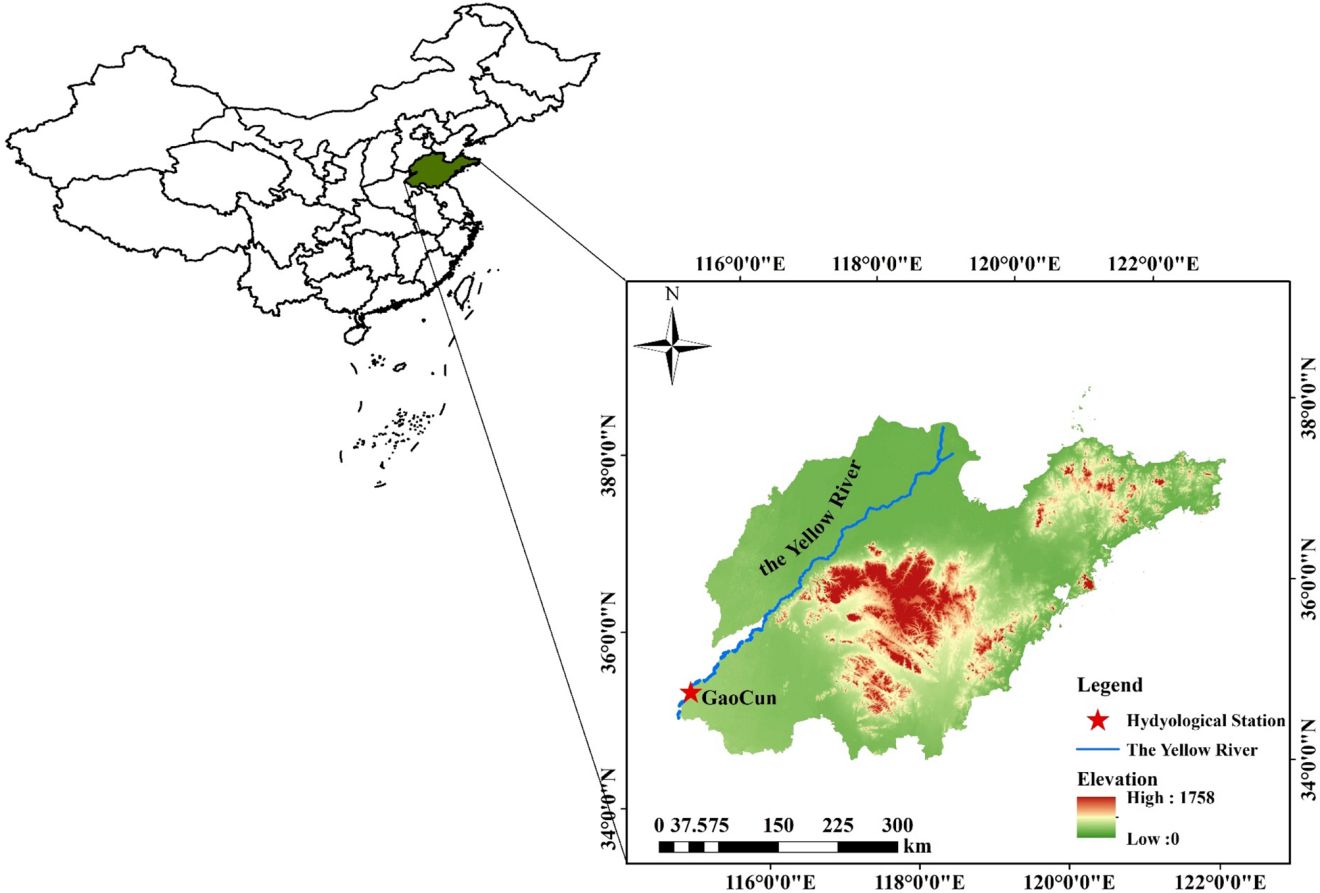
Location map of the study area.

The Yellow River basin, spanning between 96°–119° E and 32°–42° N, covers an area of approximately 79.5 × 10^4^ km^2^. It is divided into three sections: the upper, middle, and lower reaches. Over the past few decades, the measured runoff in the basin has exhibited a declining trend, accompanied by significant inter-annual variations and differences in intra-annual distribution. These challenges have resulted in severe water supply and demand issues throughout the basin. The problem of sedimentation exacerbates the conflict between water supply and demand, particularly in the downstream section. Hence, accurate runoff prediction in the lower Yellow River basin, including the Gaocun hydrological station, is crucial. Gaocun hydrological station, established in 1934 in Dongming County, Heze City, is located in the lower reaches of the Yellow River basin. It serves as the gateway station for the Yellow River’s entry into Shandong Province. The station plays a vital role in managing and developing the Yellow River, as well as in flood control, water resource management, and providing hydrological information for Shandong's social and economic development. In this article, the monthly meridional flow data of Gaocun Hydrological Station in the lower reaches of the Yellow River from 1950 to 2021 (852 months in total) were selected, and the data information was obtained from the measured data of Gaocun Hydrological Station in the lower reaches of the Yellow River, and was checked for triteness. The location of the study area is shown in Fig. 5, and this figure is created using ArcMap 10.2, URL:www.arcgis.com.

**Figure 6:**
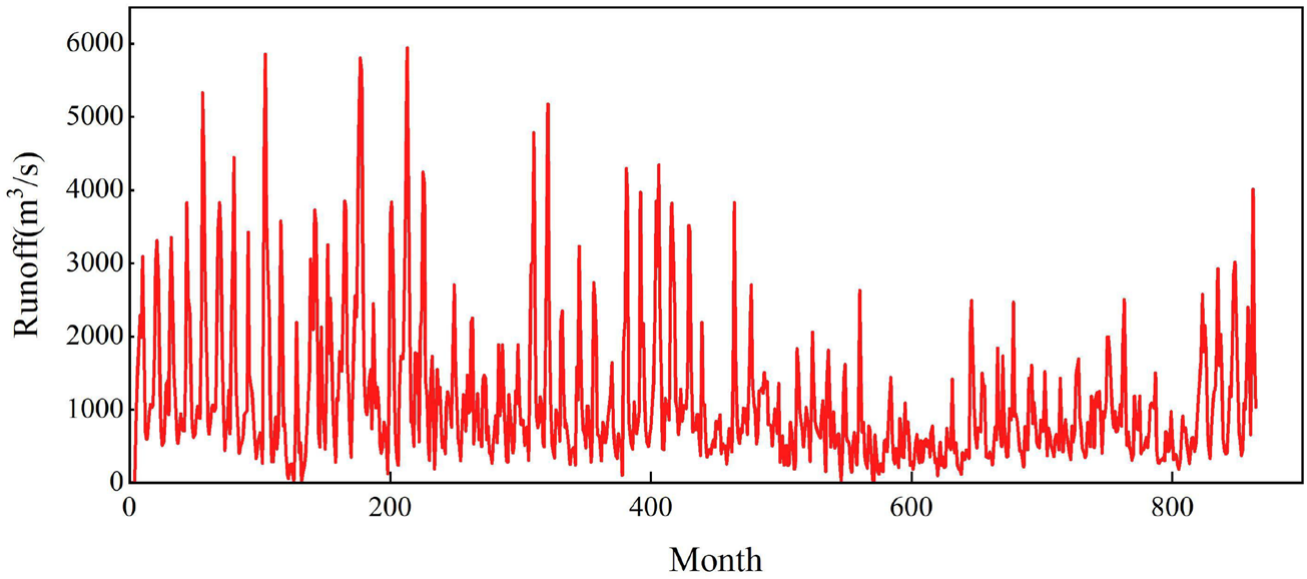
Monthly runoff series graphs.

The monthly runoff data measured at the Gaocun hydrological station from 1950 to 2021 were utilized in this study. The dataset was divided into a training set, comprising the initial 70% of the data, and a test set, comprising the remaining 30%. Figure 6 presents the graphical representation of the monthly runoff series in the study area.

### VMD decomposition

**Figure 7:**
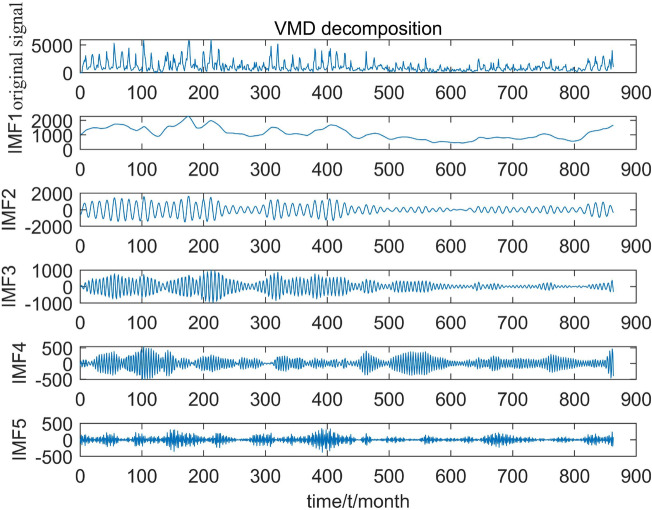
VMD decomposition diagram.

To predict the 13th month based on the inter-annual variation pattern, the first 12 months of each component were selected as inputs for the model. The selection of the number of modes (*k*) in the VMD decomposition greatly impacts the decomposition effect. A larger number of decompositions can result in frequency mixing, while a smaller number may lead to the loss of original signal information. Different *k* values were tested, and it was observed that when *k* was set to 5, the corresponding center frequencies were more dispersed. Hence,* k* = 5 was chosen. Figure 7 illustrates the decomposed subsequences with 5 different frequencies. In contrast to Fig. 6, the original runoff sequence does not exhibit a clear pattern of amplitude change. However, in Fig. 7, as the number of modal components increases, the amplitude change of the sequence becomes periodic, resulting in a more stable sequence. This preprocessing step enables the SSA-BiLSTM model to better capture the feature information in the data and make accurate predictions.

### Parameter settings

The BiLSTM model utilizes two hidden layers with 20 neurons in the first hidden layer (*H1*), 20 neurons in the second hidden layer (*H2*), 100 training iterations (*E*), and a learning rate (*η*) of 0.005. In the VMD-SSA-BiLSTM model, the sparrow population size (*N*) is set to 3, and the maximum number of iterations (*M*) is 5. Among the sparrow population, 20% are discoverers, while the rest are enrollees. The warning value is set to 0.8, indicating the presence or absence of predators. A value less than 0.8 signifies the absence of predators, while a value greater than or equal to 0.8 indicates the presence of predators, necessitating foraging in safer areas. The sparrow search algorithm explores the parameter ranges [1,100] for *H1* and *H2*, [1,100] for *E*, and [0.001,0.01] for *η* in the search for optimal BiLSTM parameters.

### Forecast results

**Figure 8:**
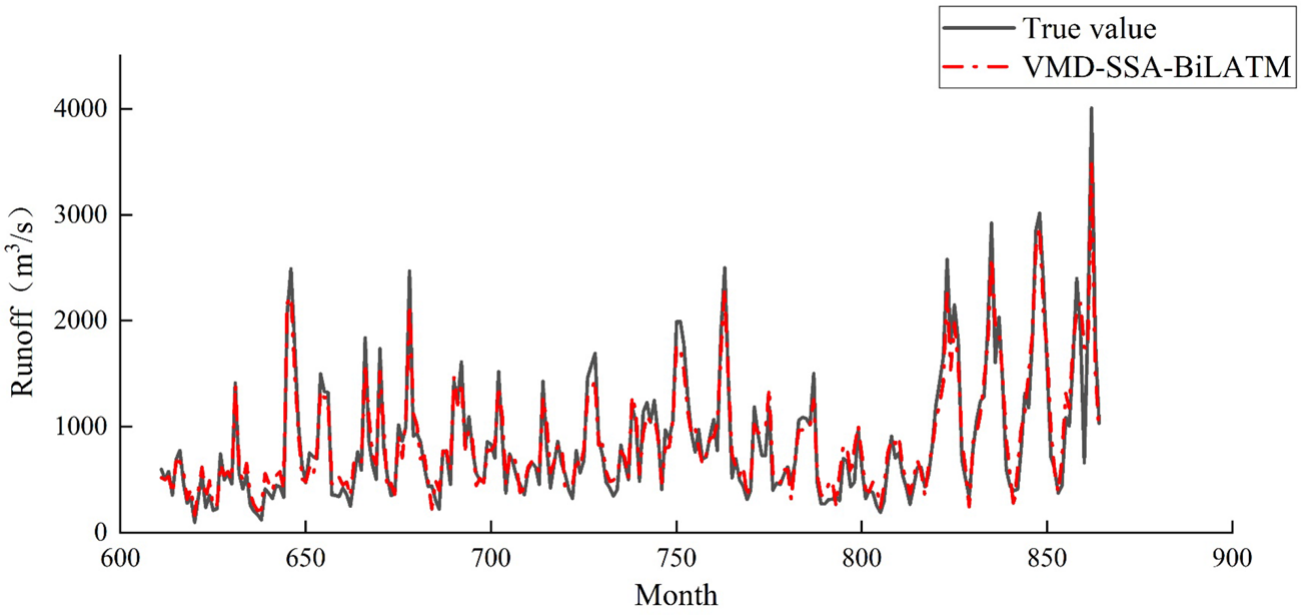
Runoff prediction curve for august.

The monthly runoff data from 1950 to 2021 at Gaocun Hydrological Station in the lower reaches of the Yellow River were used for prediction. The dataset consisted of 864 months. The first 70% of the dataset (months 1–610) was used for training, while the remaining 30% (months 611–864) was used for testing. Extensive experiments were conducted, and the obtained prediction results are presented in Fig. 8.

Based on the observation of Fig. 8, the VMD-SSA-BiLSTM coupled model shows a generally accurate prediction of monthly runoff. Although there are a few instances where the predicted values deviate significantly from the actual values, overall, the predicted values align closely with the actual values. This indicates that the model exhibits good prediction accuracy, supporting the conclusion that the model is reasonable.

### Model comparison and analysis

Four models, namely LSTM, BiLSTM, VMD-BiLSTM, and VMD-SSA-BiLSTM, were employed to predict the monthly runoff of Gaocun Hydrological Station. A statistical analysis was conducted on the error indicators, including Root-mean-square deviation (*RMSE*), mean absolute percentage error (*MAPE*), mean absolute error (*MAE*), correlation coefficient square (*R*^*2*^), and Nash coefficient (NSE) of the four models. The following are the calculation formulas for the five error indicators,

$${\text{Formula}}\;1:\;RMSE = \sqrt {\frac{1}{N} \cdot \mathop \sum \limits_{i = 1}^{N} \left( {y_{i} - p_{i} } \right)^{2} }$$

$${\text{Formula }}2:\;MAPE = \frac{100}{N} \cdot \mathop \sum \limits_{i = 1}^{N} \frac{{y_{i} - P^{i} }}{{y_{i} }},y \ne 0$$

$${\text{Formula }}3:\;MAE = \frac{1}{N}\mathop \sum \limits_{i = 1}^{N} \left| {y_{i} - p_{i} } \right|$$

$${\text{Formula }}4:\;R^{2} = \frac{SSR}{{SST}} = \frac{{\sum \left( {\widehat{{y_{i} }} - \overline{y}} \right)^{2} }}{{\sum \left( {y_{i} - \overline{y}} \right)^{2} }}$$

$${\text{Formula }}5:\;ESE = 1 - \frac{{\mathop \sum \nolimits_{t = 1}^{T} \left( {Q_{o}^{t} - Q_{m}^{t} } \right)^{2} }}{{\mathop \sum \nolimits_{t = 1}^{T} \left( {Q_{o}^{t} - \overline{{Q_{o} }} } \right)^{2} }}$$

The results of the analysis are presented in Table 1, as depicted below.

**Table 1:** Parameter rate and test performance statistics for the four models.

	Models	LS™	BiLS™	VMD-BiLS™	VMD-SSA-BiLS™
Training period	RMSE	395.6334	311.0775	88.6966	37.7533
	MAPE/%	56.6173	48.3651	14.7437	5.6832
	MAE	213.64	173.51	46.7185	19.8992
	R2	0.12118	0.39017	0.77656	0.93775
	NSE	0.3523	0.4423	0.8364	0.9717
Validation period	RMSE	321.5514	273.1725	76.6436	30.6601
	MAPE/%	48.4025	40.2843	11.0762	4.6906
	MAE	197.6471	151.9632	40.9618	15.2344
	R2	0.14662	0.41312	0.79632	0.94371
	NSE	0.3964	0.4892	0.8764	0.9886

Table 1 presents the results of the analysis, revealing that the BiLS™ model outperforms the LS™ model in predicting monthly runoff. During the validation period, the *RMSE* index decreases by 48.3789, the *MAPE* index decreases by 8.1182%, the *MAE* index decreases by 45.6839, the *R2* index increases by 0.2665, and the *Nash* index increases by 0.0928. Hence, BiLS™ is selected as the basis for model construction in this study.

Furthermore, the VMD-BiLS™ model, formed by incorporating VMD decomposition and reconstruction into the BiLS™ model, yields more accurate prediction results. Additionally, the VMD-SSA-BiLS™ model, optimized using the sparrow search algorithm (SSA), demonstrates significant advantages over the first two models during the retraining and validation periods. Specifically, the *RMSE* index decreases by 242.5124 and 45.9835, the *MAPE* index decreases by 35.5937% and 6.3856%, the* MAE* index decreases by 136.7288 and 25.7274, the *R2* index increases by 0.53059 and 0.14739, and the *NSE* index increases by 0.4994 and 0.1122, respectively.

Based on the comparative analysis of the error metrics, it is evident that the VMD-SSA-BiLS™ model exhibits the highest prediction accuracy.

## Discussion

**Figure 9:**
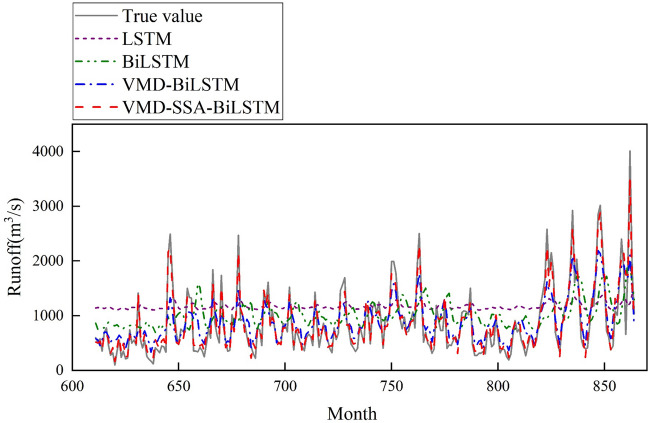
Comparison of the prediction process of the four models during the validation period.

**Figure 10:**
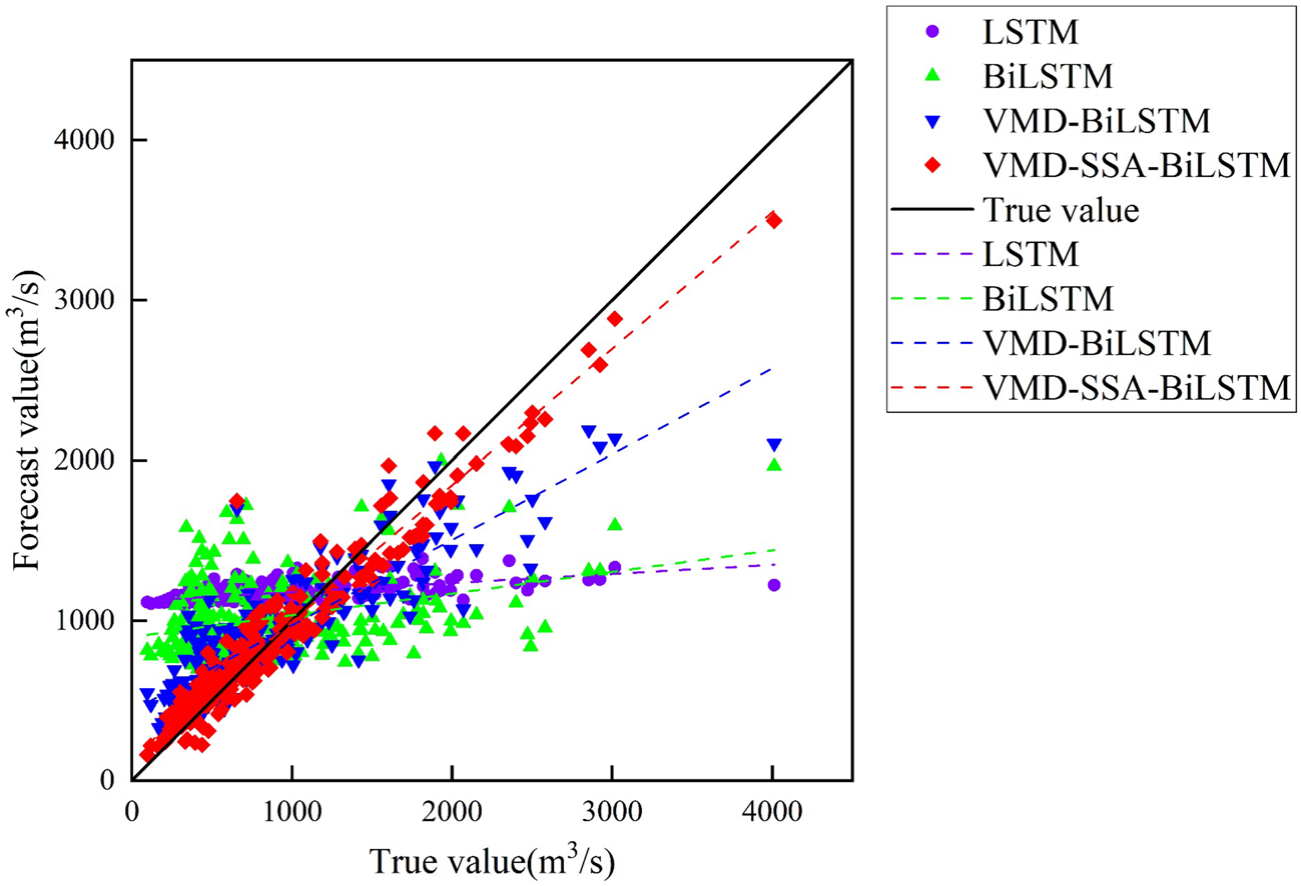
Scatter plot of predicted and measured monthly runoff for the validation period of the four models.

**Figure 11:**
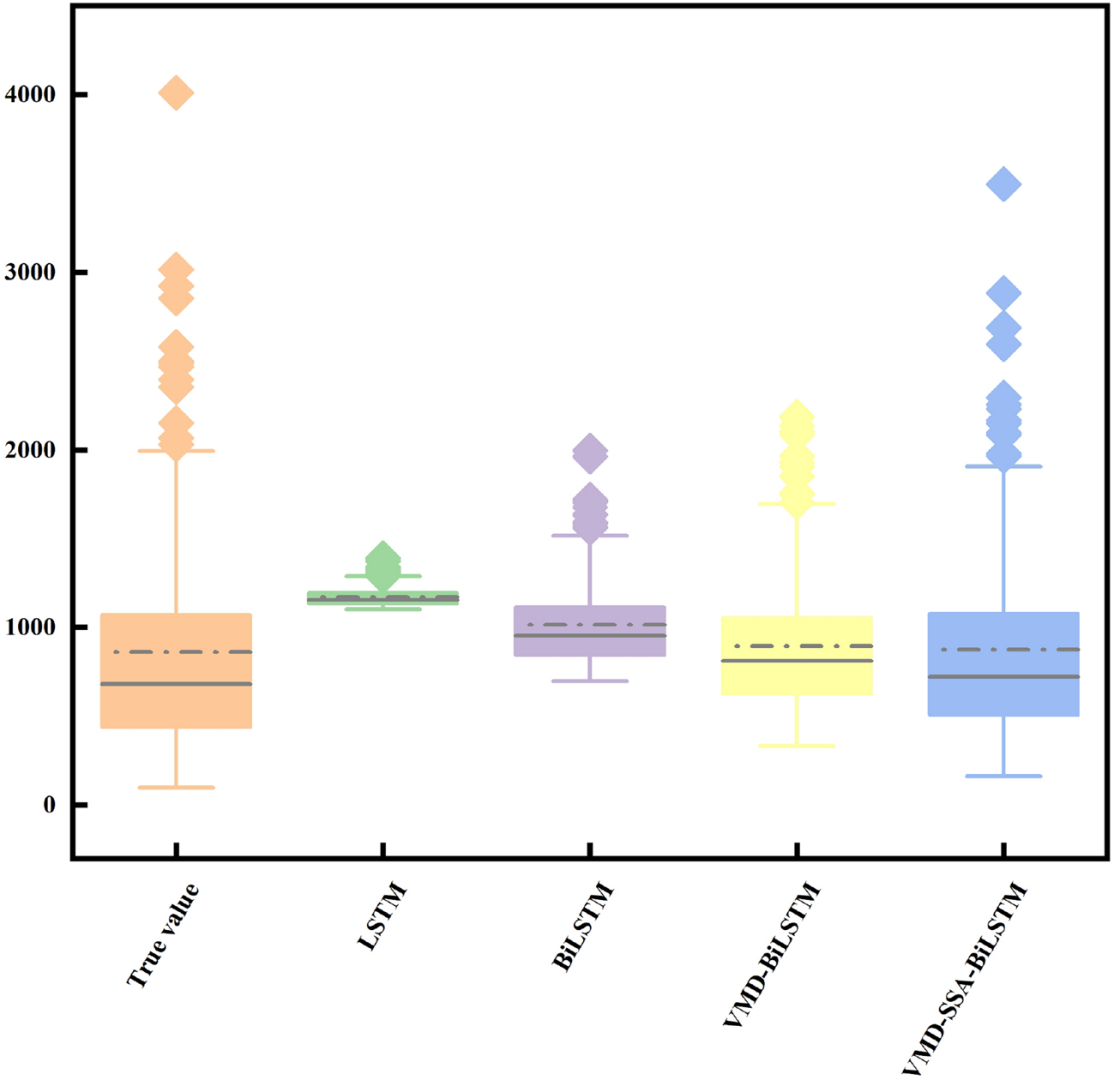
Box plot of predicted and measured monthly runoff for the validation period of the four models.

**Figure 12:**
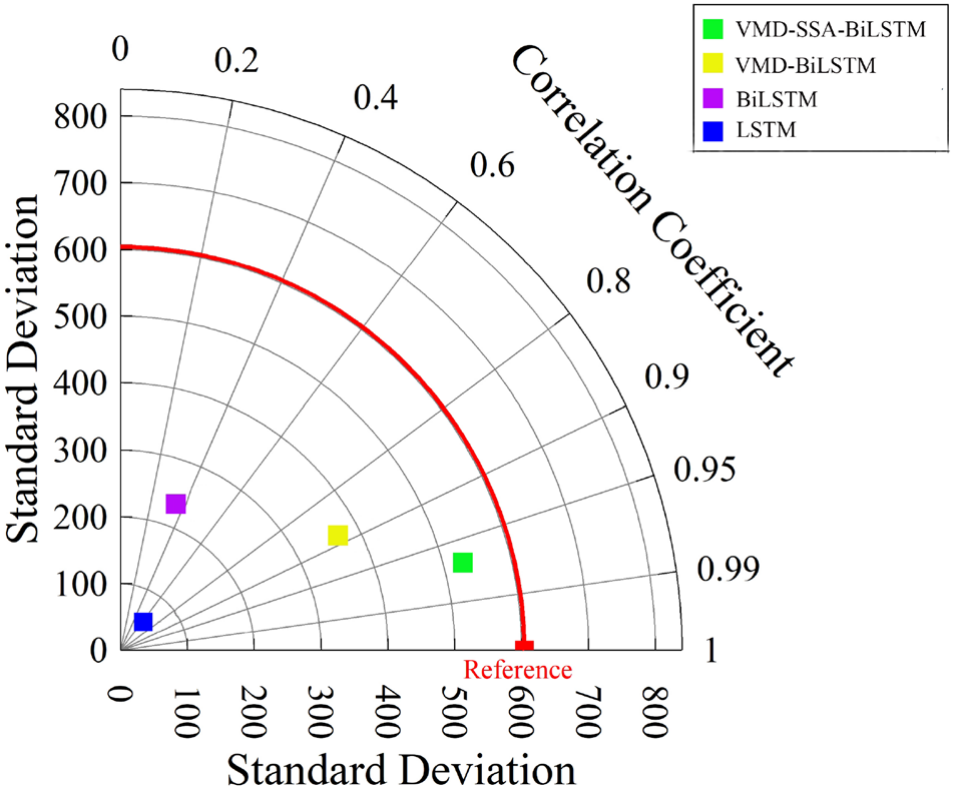
Taylor diagram of predicted and measured monthly runoff for the validation period of the four models.

From Fig. 9, it can be seen that the predicted results of monthly runoff using the LS™ model and BiLS™ model alone differ significantly from the measured data, resulting in poor prediction performance. However, after comparing the two, it can be concluded that the BiLS™ model has slightly better prediction performance than LS™; However, the predicted values obtained by using VMD-BiLS™ and VMD-SSA-BiLS™ models are relatively consistent with the measured values, but it is difficult to distinguish which model has the better prediction effect based on Fig. 9 alone; Furthermore, according to the scatter plot in Fig. 10, it can be seen that the prediction accuracy of the VMD-SSA-BiLS™ model is slightly better than that of VMD-BiLS™. Figure 11 shows the Box plot drawn from the model prediction data and the measured data. It can be seen from the graphic comparison that the LS™ model used alone has the worst prediction effect, while the VMD-SSA-BiLS™ model has the best prediction effect. In addition, it can be seen from the Taylor diagram in Fig. 12 that the prediction accuracy of the VMD-SSA-BiLS™ model is the highest.In summary, both the single LS™ model and the BiLS™ model are not effective in predicting monthly runoff. This may be due to the fact that a single time series direct prediction cannot take into account the physical conditions of the basin, considering that the upstream of the Gaocun hydrological station will be influenced by other large and small hydropower stations on runoff, and the mechanism of runoff formation is relatively complex, making the single prediction model less accurate and unable to meet the actual prediction needs. The VMD decomposition can reduce the noise of the original runoff series and extract the complex and effective information implied in the runoff data, which to a certain extent can reflect the intrinsic mechanism of watershed runoff formation. The SSA further optimizes the BiLS™ model to obtain the optimal parameters for the number of hidden units, the maximum training period and the initial learning rate, which improves the efficiency of the model parameter selection. The coupled VMD-SSA-BiLS™ model can achieve high prediction accuracy in both the training and validation periods, which shows that the VMD-SSA-BiLS™ model proposed in this paper is feasible for the prediction of monthly runoff. In comparison with other research articles on runoff prediction, this paper firstly addresses the relatively complexity of runoff formation mechanisms in the study area by performing data noise reduction prior to parameter optimisation.

## Conclusions

1. This study proposes a monthly runoff prediction model based on the coupling of VMD-SSA-BiLS™, which combines variational mode decomposition (VMD), sparrow search algorithm (SSA), and bidirectional Long short-term memory neural network (BiLS™). Compared with previous studies on runoff prediction, this paper constructs a coupled VMD-SSA-BiLS™ model, which combines the advantages of the three and improves the prediction accuracy; at the same time, the BiLS™ neural network, which has been less applied in runoff prediction previously, is used as one of the research methods, and good results have been achieved. The model effectively addresses the nonstationarity of monthly runoff series and enhances the accuracy of point prediction. Additionally, the nonparametric Kernel density estimation provides prediction intervals for monthly runoff without assuming error distribution in advance. This compensates for the limitations of point prediction models in describing prediction results and offers a novel approach to monthly runoff prediction.
2. The model was applied to predict monthly runoff at the Takamura hydrological station. The obtained results include a root mean square error (*RMSE*) of 30.6601, a mean absolute percentage error (*MAPE*) of 4.6906%, a mean absolute error (*MAE*) of 15.2344, a mean correlation coefficient squared (*R2*) of 0.94371, and a Nash coefficient (*NSE*) of 0.9886.
3. The VMD-SSA-BiLS™ coupled model fully utilizes the benefits of VMD decomposition for data noise reduction and the SSA optimization algorithm for optimizing BiLS™ neural network parameters. Compared to the BiLS™ model and the VMD-BiLS™ model, the VMD-SSA-BiLS™ model achieves notable improvements. Specifically, the *RMSE* is reduced by 242.5124 and 45.9835, the *MAPE* is reduced by 35.5937% and 6.3856%, the *MAE* is reduced by 136.7288 and 25.7274, the *R2* is increased by 0.53059 and 0.14739, and the *NSE* is increased by 0.4994 and 0.1122, respectively. In this paper, only a single-step prediction of the monthly runoff series during the flood season is presented. The sparrow optimisation algorithm has better optimisation performance than the traditional population optimisation algorithm, but still suffers from too fast convergence and the tendency to fall into local optima. The advantage of the model proposed in this article lies in the idea of "decomposition-prediction-reconstruction", which improves the final prediction accuracy with each step of model construction. Then, the BiLS™ model used for prediction, which has the advantages of bidirectional link and advanced characteristics, is also very suitable for runoff prediction. In addition, regarding the limitations of the model, the sparrow optimization algorithm has the problem of too fast convergence for parameter optimization, which can easily result in only local optima. This model has good performance in predicting single time series runoff data, but further improvement is needed when applied to predicting multiple series data.
4. In this paper, compared with other research articles, the more novel BiLS™ model was chosen for the prediction method, and in terms of data processing, the VMD decomposition method was used for noise reduction of the data, together with the SSA optimisation algorithm coupled with the model, to obtain a high prediction accuracy.
5. In this paper, only a single-step prediction of the monthly runoff series during the flood season is presented. In subsequent studies, the proposed model can be used to make further multi-step predictions on the basis of the single-step prediction. The sparrow optimisation algorithm has better optimisation performance than the traditional population optimisation algorithm, but still suffers from too fast convergence and the tendency to fall into local optima. In the future, suitable algorithms should also be selected for forecasting for the selected study area, further achieving extended research from algorithm improvement and model construction. For future scope of the model, the model can be used for further multi-step prediction based on single step prediction. Also it can be tried to use this model in other predictions such as annual runoff prediction, seasonal runoff prediction.

## Data Availability

Data and materials are available from the corresponding author upon request.
